# Mechanism of isoleucyl-tRNA synthetase 2 regulating proliferation and apoptosis of cervical cancer cells

**DOI:** 10.1038/s41598-026-41218-7

**Published:** 2026-03-02

**Authors:** Yuxin Bi, Yuqin Ye, Xufeng Wu, Huang Cao, Quanfu Ma

**Affiliations:** 1 Maternal and Child Health Hospital of Hubei Province, Huazhong University of Science and Technology, Wuhan, China; 2 Medical College of Wuhan University of Science and Technology, Wuhan, China; 3 Hubei Clinical Medical Research Center for Gynecologic Malignancy, Wuhan, China; 4 Hubei Provincial Center for Medical Genetics, Wuhan, China; 5 Zhongnan Hospital of Wuhan University, Wuhan, China

**Keywords:** IARS2, mTOR, Rag, eIF4E, Cervical cancer, Cancer, Cell biology, Molecular biology, Oncology

## Abstract

Isoleucyl-tRNA synthetase 2 (IARS2) is a mitochondrial isoleucine-tRNA synthetase of the class I aminoacyl-tRNA synthetase family, and its role in cervical cancer remains poorly defined. Here, we report that IARS2 promotes cervical cancer cell proliferation and survival through regulation of the Rag GTPases and mTORC1. Elevated IARS2 expression was associated with poorer overall survival in patients with cervical cancer. Silencing of IARS2 significantly reduced cell viability and induced apoptosis in cervical cancer cells. Mechanistically, IARS2 inhibited mTOR degradation and sustained activation of the mTOR–eIF4E axis, thereby supporting cell proliferation. The amino acid–binding pocket of IARS2 was essential for these regulatory effects. Moreover, immunoprecipitation assays revealed that IARS2 interacted with Rag GTPases, particularly RagB and RagD. Together, these findings suggest that IARS2 may function as an amino acid sensor within the Rag–mTORC1 complex and highlight IARS2 as a potential therapeutic target in cervical cancer.

**Supplementary Information:**

The online version contains supplementary material available at 10.1038/s41598-026-41218-7.

## Introduction

Aminoacyl-tRNA synthetases (ARSs), which possess both canonical aminoacylation activity and the ability to sense intracellular amino acid availability, have been increasingly recognized as important contributors to cancer development and progression^1–3^. Mitochondrial aminoacyl-tRNA synthetases (mtARSs), a distinct subgroup of ARSs, are primarily responsible for mitochondrial protein synthesis^4^. Accumulating evidence has revealed that dysregulation of mtARSs is closely associated with tumorigenesis and cancer progression^5,6^.

Isoleucyl-tRNA synthetase 2 (IARS2) is a nuclear-encoded mitochondrial isoleucine-tRNA synthetase belonging to the class I aminoacyl-tRNA synthetase family^7^. It catalyzes a two-step aminoacylation reaction involving ATP-dependent activation of isoleucine followed by its esterification to the 3′ end of the cognate tRNA^8^. IARS2 contains two highly conserved signature motifs, the “HIGH” and “KMSKS” sequences^9^. The HIGH motif functions as an ATP-binding site, whereas the KMSKS motif is critical for amino acid transfer to tRNA^10^. Pathogenic mutations in the IARS2 gene have been implicated in mitochondrial disorders^11^. IARS2 harbors a mitochondrial targeting sequence that facilitates its translocation into mitochondria, and it is generally accepted that IARS2 is synthesized in the cytoplasm and exerts its canonical function within mitochondria. Notably, recent studies have suggested that IARS2 may act as a cancer-promoting factor in multiple malignancies^2,12–14^. For instance, Xin Di et al. reported that knockdown of IARS2 significantly suppressed AKT/mTOR signaling in non-small cell lung cancer (NSCLC)^13^, while Hong Li et al. demonstrated that IARS2 depletion inhibited proliferation of acute myeloid leukemia HL-60 cells through regulation of the p53/eIF4E pathway^15^.

The mechanistic/mammalian target of rapamycin (mTOR) is a central regulator of cellular metabolism, growth, and survival^16^. Aberrant activation of mTOR signaling has been implicated in numerous diseases, including cancer, obesity, diabetes, and neurodegenerative disorders^16^. In mammalian cells, mTOR assembles into two distinct multiprotein complexes, mTOR complex 1 (mTORC1) and mTOR complex 2 (mTORC2), which mediate different cellular functions^17^. Among them, mTORC1 is highly sensitive to nutrient availability, particularly amino acids^18^. According to the consensus model of amino acid-dependent mTORC1 activation, leucine (Leu), arginine (Arg), and S-adenosylmethionine (SAM) are sensed by cytosolic and lysosomal amino acid sensors^19^. These signals converge on Ras-related GTP-binding (Rag) GTPases, which recruit mTORC1 to the lysosomal surface for activation^17^. Rag belongs to one GTPase that hydrolyzes GTP to GDP^20,21^. They operate on the lysosome surface where they sense amino acid levels from the cytosol and lysosomal lumen^22^, and this is necessary for the activation of mTORC1 pathway^23^. There are four isoforms of Rag, namely RagA, RagB, RagC, and RagD. These Rag proteins must form dimers (RagA-C or RagB-D) to exert their functional roles^20,21^. Heterodimers composed of RagA/B^GTP^-RagC/D^GDP^ form during amino acid sufficiency to promote mTORC1 signaling and heterodimers composed of RagA/B^GDP^-RagC/D^GTP^ form during amino acid depletion to inactive mTORC1 signaling^23,24^. Kezhen Huang concluded that the nucleotide-bound state of RagC/D as well as that of RagA/B indeed contributes to mTORC1 signaling in response to amino acids^25^.Recent studies have further revealed that certain ARSs, including leucyl-tRNA synthetase (LARS) and mitochondrial threonyl-tRNA synthetase 2 (TARS2), can function as intracellular amino acid sensors that directly regulate mTORC1 activity^10,26^. One of the principal functions of mTORC1 is to promote protein synthesis by enhancing mRNA translation and ribosomal biogenesis^17^. This effect is largely mediated through phosphorylation of eukaryotic initiation factor 4E-binding protein 1 (4E-BP1), which releases and activates eukaryotic initiation factor 4E (eIF4E), the rate-limiting factor for cap-dependent translation^27^.

Cervical cancer (CC) is one of the most common gynecological malignancies worldwide^28^. Concurrent chemoradiotherapy remains the standard treatment for advanced cervical cancer^29^. However, therapeutic outcomes are often limited, partly due to restricted access to radiotherapy and treatment resistance^30^. Therefore, identification of novel molecular targets and therapeutic strategies for cervical cancer is urgently needed. In this study, we elucidated that IARS2 regulates the proliferation and apoptosis of cervical cancer by Rags-mTORC1, preventing the degradation of mTOR.

## Materials and methods

### Cell culture and transient transfection

The human cervical cancer cell line HeLa was kindly provided by Prof. Hongbing Shu and maintained in Dulbecco’s modified Eagle medium (DMEM; CellMax, CGM314.05) supplemented with 2 mM Ala-Gln (OKA, D10184, 0.2 μm PES-filtered) and 10% fetal bovine serum (FBS; CellMax, SA211.02). Cells were cultured at 37 °C in a humidified incubator containing 5% CO₂. Antibiotics were not routinely added unless otherwise specified. Transient transfection of plasmids and/or small interfering RNAs (siRNAs) was performed using Lipofectamine 3000 (Invitrogen, L3000015) or Lipo8000 (Beyotime, C0533) according to the manufacturers’ instructions. For each construct, three independent transfection experiments were conducted using freshly prepared cells. Protein expression was analyzed by western blotting.

### Plasmids

The pcDNA3.1(–) vector was used as the backbone for plasmid construction. All inserts or modified regions generated during molecular cloning were verified by Sanger sequencing (Sangon Biotech, Shanghai, China), whereas plasmid backbones and unmodified regions were not sequenced. Seamless cloning (Bio Basic Inc., B632219) was employed for constructing IARS2 mutant plasmids. Multiple sequence alignment of IARS2 key residues mutations was performed using the Constraint-based Multiple Alignment Tool (COBALT)^31,32^. Primer sequences used for plasmid construction are listed in Supplementary Information 1, Table S1. All plasmids generated in this study were deposited at Addgene ( https://www.addgene.org/browse/article/28252609/ ) and are also described in Supplementary information3. Addgene identification numbers for individual plasmids are provided in Supplementary Information 1, Table S2. Seamless cloning was performed using the Seamless Cloning Master Mix (Bio Basic Inc., B632219-0040), and traditional cloning was conducted using the DNA Ligation Kit < Mighty Mix> (TaKaRa, 6023). Restriction enzymes used included SalI (TaKaRa, 1080 S), XhoI (TaKaRa, 1094 S), EcoRI (TaKaRa, 1040 S), and HindIII (TaKaRa, 1060 S). Plasmids pCMV-RRAGA-3×FLAG-Neo (P45036), pCMV-RRAGB-3×FLAG-Neo (P55832), pCMV-RRAGC-3×FLAG-Neo (P43680), and pCMV-RRAGD-3×FLAG-Neo (P55833) were purchased from MiaoLing Bio (Wuhan, China).

### RNA interference

SiRNAs were synthesized by Sangon Biotech. For gene silencing, 400 pmol of siRNA was transfected per well of a six-well plate. Due to the high endogenous abundance of IARS2, a higher siRNA concentration than commonly used was required. The sequence of siIARS2 (sense strand) was 5′-ACUUGCAGUCAUCCAUUAA-3′. Knockdown efficiency was confirmed by western blotting. OPTI-MEM (Thermo Fisher Scientific, 31985062) was used as the transfection medium and replaced with complete medium 6–8 h after transfection.

### Western blotting and co-immunoprecipitation

For western blotting, cells were lysed in 2× Laemmli buffer supplemented with β-mercaptoethanol (2%), dithiothreitol (DTT; 2 mM), and phenylmethylsulfonyl fluoride (PMSF; 0.1 mM). Protein concentrations were determined using a modified Bradford assay^33,34^. Equal amounts of protein (20 µg per sample) were separated by 8% SDS–PAGE and transferred onto 0.45 μm PVDF membranes (Merck Millipore, Germany). Membranes were blocked with 5% skim milk at room temperature for 2 h and incubated overnight at 4 °C with primary antibodies against IARS2 (Proteintech, 17170-1-AP; 1:500), eIF4E (Proteintech, 66655-1-Ig; 1:10,000), mTOR (Proteintech, 66888-Ig; 1:5,000), TOM20 (Proteintech, 11802-1-AP; 1:2,000), and MTCO2 (Bio Basic Inc., D160007; 1:1,000). For detection of phosphorylated mTOR (Ser2448), membranes were blocked using a protein-free blocking reagent (Sangon Biotech, C530040) and incubated with anti–phospho-mTOR antibody (ZEN-BIOSCIENCE, 381557; 1:1,000). After incubation with appropriate HRP-conjugated secondary antibodies, signals were visualized using an enhanced chemiluminescence detection kit (BL520A; Biosharp, China). Densitometric analysis was performed using ImageJ^35^, and and protein levels were normalized to actin.

Co-immunoprecipitation (co-IP) was performed as previously described^36^. Briefly, HeLa cells were transfected in 10-cm dishes with 30 µg of pCMV-RRAGA-3×FLAG-Neo, pCMV-RRAGB-3×FLAG-Neo, pCMV-RRAGC-3×FLAG-Neo, or pCMV-RRAGD-3×FLAG-Neo plasmids and cultured for 48 h. Cells were lysed in IP lysis buffer containing protease inhibitors, and lysates were clarified by centrifugation at 13,800 × g for 30 min at 4 °C. Protein concentrations were measured, and equal amounts of lysates were incubated with mouse anti-FLAG antibody or control mouse IgG at 4 °C overnight, with 5% of each lysate reserved as input. Protein A/G agarose beads were then added and incubated for an additional 4 h. Immunoprecipitated proteins were analyzed by western blotting using the indicated antibodies.

The following antibodies were used for IP and co-IP analysis: anti-FLAG (F1804; Sigma) antibody and Normal mouse IgG (Bio Basic Inc, D110503-0001) antibody. Note that for co-IP assay in this study, each of the plasmids was transfected individually without co-transfection of IARS2 plasmid, and endogenous IARS2 co-precipitated was detected by WB.

### Cycloheximide chasing assay

HeLa cells were transfected with siCtrl or siIARS2 as described above. At 24 h post-transfection, the cells were treated with cycloheximide (CHX; 100 µg/mL). Total proteins were collected at 0, 4, 12, 24, and 48 h after CHX treatment and analyzed by western blotting.

### Cell counting kit-8 assay

Cell viability was evaluated using the Cell Counting Kit-8 (CCK-8) assay. HeLa cells were transfected with the indicated siRNAs and/or plasmids and reseeded into 96-well plates at a density of 2,000 cells per well 24 h post-transfection. Cell viability was measured at 24, 48, and 72 h after seeding. Seven technical replicates were included for each group at each time point. Statistical significance was determined by Student’s t-test, with *P* < 0.05 considered significant and indicated by an asterisk (*).

### Caspase3/7 Glo assay

Caspase-3/7 activity was measured using the Caspase-Glo^®^ 3/7 assay (Promega, #72052). Three independent biological replicates were performed. In each replicate, HeLa cells were transfected in 24-well plates with the indicated plasmids and siRNAs. At 24 h post-transfection, culture medium was removed, and 100 µL PBS plus 100 µL Caspase-Glo reagent were added to each well. Plates were shaken for 30 min at room temperature, and luminescence was measured using a luminometer (BLT, Lux-T020). Four technical replicates were included per condition. Statistical significance was assessed by Student’s t-test (*P* < 0.05).

### Annexin V apoptosis assay

Apoptosis was analyzed using an Annexin V–FITC/PI apoptosis detection kit (Servicebio, G1511). HeLa cells were transfected with siIARS2 or siCtrl, harvested using EDTA-free trypsin, washed with cold PBS, and stained according to the manufacturer’s instructions. Apoptotic cells were quantified by flow cytometry (Beckman CytoFLEX).

### RNA extraction and quantitative real-time PCR

Total RNA was extracted from HeLa cells 48 h after transfection using RNA Extraction Solution (Servicebio, G3013) according to the manufacturer’s instructions. The experiment was performed with three independent biological replicates from separate transfections. cDNA synthesis was performed using SweScript All-in-One RT SuperMix (Servicebio, G3337), and quantitative real-time PCR was conducted with SGExcel UltraSYBR Master Mix (Sangon Biotech, B532957) on a Bio-Rad CFX Connect system, according to the manufacturers’ instructions. Gene expression levels were normalized to GAPDH and calculated using the 2^−ΔΔCq^ method. Statistical significance was determined by Student’s t-test (*P* < 0.05). Primer sequences were shown in Supplementary Information 1, Table S3.

### Survival analysis

Overall survival analysis was performed using the Gene Expression Profiling Interactive Analysis (GEPIA) web tool (https://GEPIA.cancer-PKU.cn/), based on data from The Cancer Genome Atlas (TCGA) database^37^.

## Results

### High expression of IARS2 is associated with poor survival in cervical cancer

Previous studies have demonstrated that IARS2 regulates proliferation and apoptosis in multiple cancer types^12,13,15,38,39^. To evaluate the effect of IARS2 on cervical cancer cells, flow cytometric analysis was performed following IARS2 silencing in HeLa cells. The results showed a significant increase in apoptotic cells in the siIARS2 group compared with the control group (Fig. S1, *P* < 0.05). However, flow cytometric analysis showed that the proportions of early apoptotic cells were relatively low in both the control and experimental groups; therefore, we subsequently employed a caspase-3/7 activity–based assay to assess apoptosis.

**Fig. 1:**
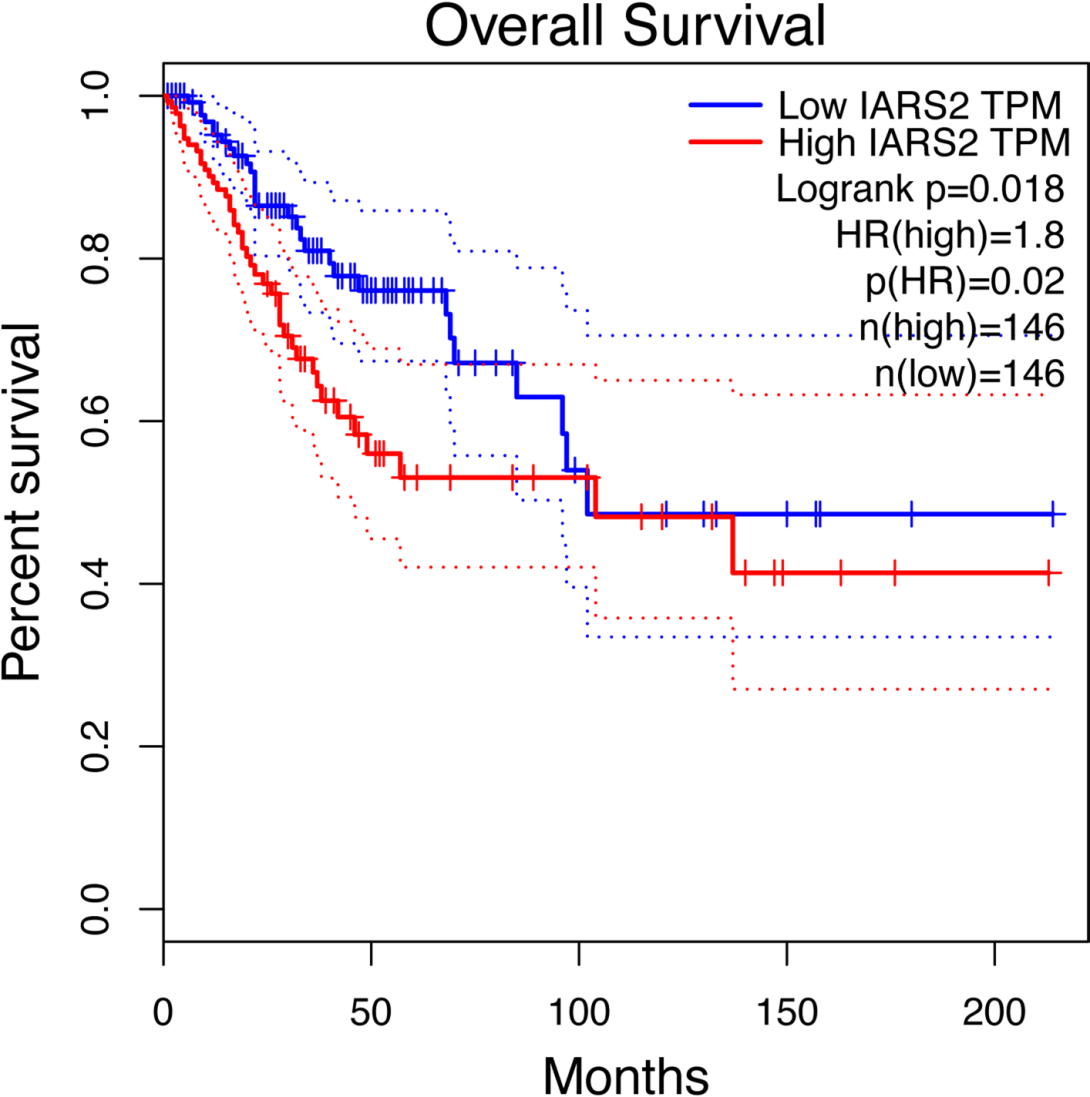
Survival plots of high IARS2 and low IARS2 patients. The red line represents patients with high expression of IARS2, and the blue line represents patients with low expression of IARS2. In the survival curve, the survival rate of people with high expression of IARS2 was 1.8 times lower compared to those with low expression.

To further assess the clinical relevance of IARS2 expression in cervical cancer, survival analysis was conducted using the GEPIA web tool based on data from The Cancer Genome Atlas (TCGA) database^37^. Patients with cervical squamous cell carcinoma (CESC) exhibiting high IARS2 expression showed significantly lower overall survival within five years compared with those with low IARS2 expression (Fig. 1, *P* < 0.05). These results indicate that elevated IARS2 expression is associated with poor prognosis in cervical cancer.

### Knockdown of IARS2 inhibits proliferation and promotes apoptosis through mTOR and eIF4E

**Fig. 2:**
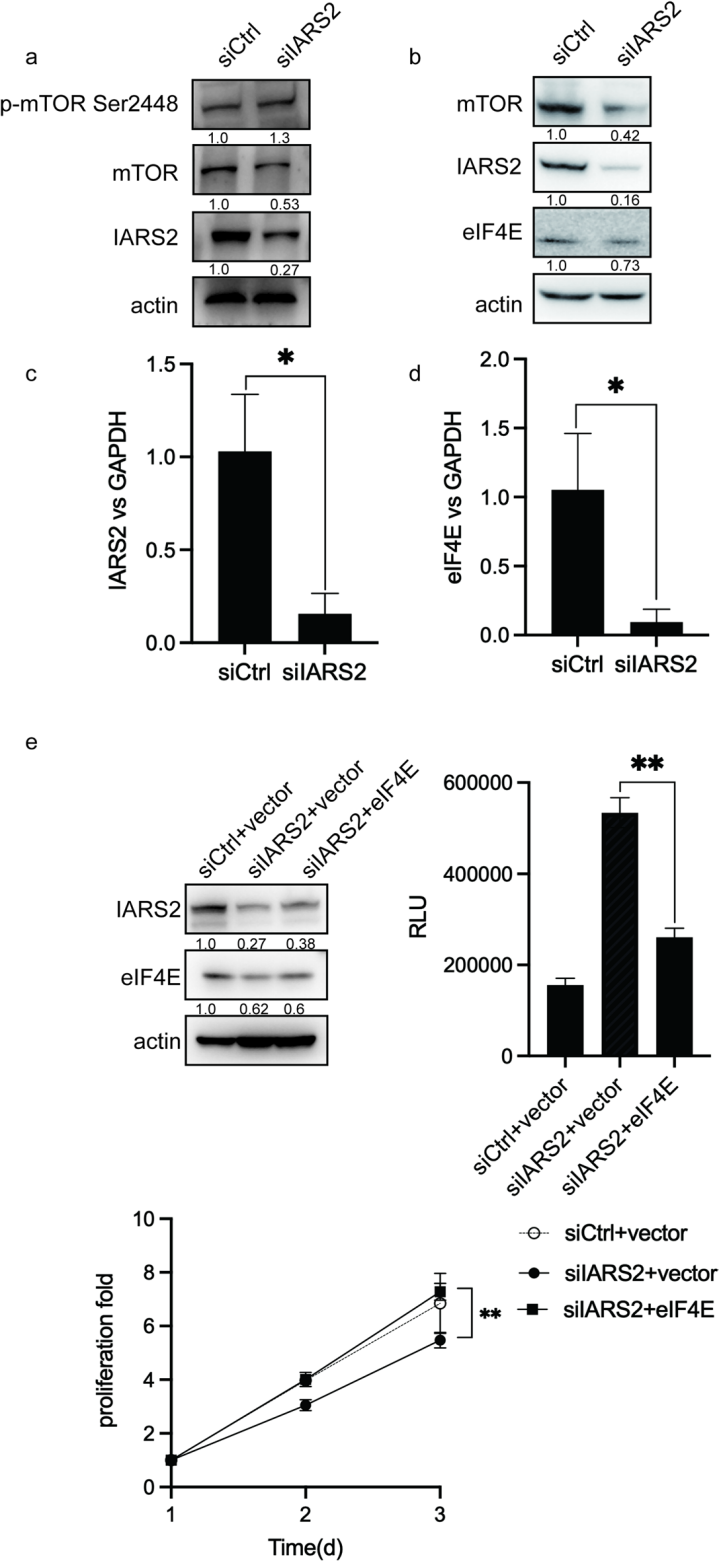
Impact of knockdown IARS2 on proliferation, apoptpsis, and mTOR/eIF4E. (**a**) The protein level of mTOR, p-mTOR Ser2448 and IARS2 in siCtrl group and siIARS2 group. The numbers below the protein images represent the densitometric ratio of each protein to actin, normallized by siCtrl. (**b**) The protein level of mTOR, IARS2, eIF4E in siCtrl group and siIARS2 group. The numbers below the protein images represent the densitometric ratio of each protein to actin, normallized by siCtrl. (**c-d**) The mRNA level of IARS2 and eIF4E in siCtrl group and siIARS2 group. (**e**) The protein level, CCK8 and caspase3/7 activity of eIF4E rescue assay. The numbers below the protein images represent the densitometric ratio of each protein to actin, normallized by siCtrl+Vector. Vector presents empty carrier pcDNA3.1(-). The Y-axis of CCK8 represents normalized OD450. The Y-axis of represents luminescence of Caspase3/7 Glo.

Western blot analysis revealed that silencing of IARS2 markedly reduced total mTOR protein levels, whereas phosphorylation of mTOR at Ser2448 was not significantly decreased in siIARS2-treated cells compared with controls (Fig. 2a, Fig. S2**a**). These findings indicate that IARS2 primarily affects total mTOR abundance rather than its phosphorylation status. Given the central role of mTORC1 in translational regulation, we next examined downstream effectors involved in protein synthesis. Among these, eukaryotic initiation factor 4E (eIF4E) was selected for further investigation due to its well-established role in mTORC1-mediated translational control and suitability for rescue experiments.

Following transfection of HeLa cells with siIARS2, cell viability was significantly reduced compared with the siCtrl group, accompanied by decreased protein levels of IARS2 and mTOR (Fig. 2b). Consistently, flow cytometric analysis showed a significant increase in apoptosis in siIARS2-treated cells (Fig. S1, *P* < 0.05). In parallel, eIF4E protein expression was also reduced upon IARS2 knockdown (Fig. 2b). RT–qPCR analysis further demonstrated that eIF4E mRNA levels were significantly decreased in the siIARS2 group (Fig. 2c–d).

To evaluate whether eIF4E functionally contributes to the cellular effects induced by IARS2 depletion, HeLa cells were co-transfected with an eIF4E overexpression plasmid together with siIARS2 or siCtrl. Restoration of eIF4E expression significantly rescued cell proliferation and attenuated apoptosis in siIARS2-treated cells (Fig. 2e, Fig. S2**e**). Collectively, these findings suggest that reduced eIF4E activity is a major downstream consequence of IARS2 knockdown and that the mTOR–eIF4E axis mediates, at least in part, the effects of IARS2 on cervical cancer cell proliferation and survival.

### Knockdown of IARS2 accelerates mTOR protein degradation in HeLa cells

Given that IARS2 knockdown reduced total mTOR protein levels without affecting mTOR phosphorylation, we hypothesized that IARS2 may regulate mTOR protein stability. Previous studies on LARS and TARS2 have focused primarily on downstream mTOR signaling without addressing mTOR protein turnover^10,26^.

**Fig. 3:**
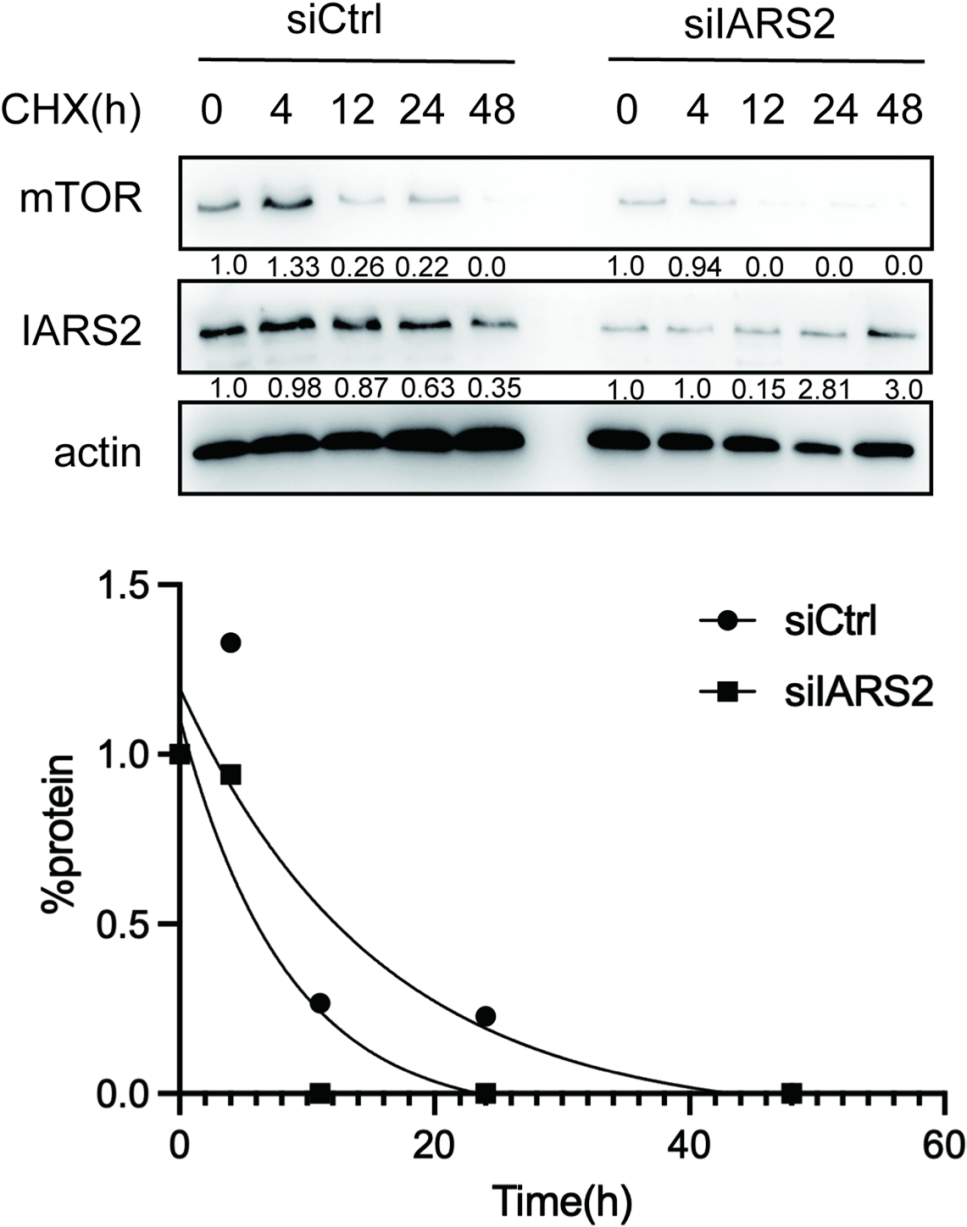
Analysis of mTOR degradation by cycloheximide chasing assay. (**a**) The protein level of mTOR and IARS2 in siCtrl group and siIARS2 group by cycloheximide chasing assay. (**b**) Normalized densitometric quantification of the mTOR protein. The %protein in line graph represents the ratio of mTOR at indicated time points and mTOR at 0 h in order to entail the initial point consistent.

To test this hypothesis, a cycloheximide (CHX) chase assay was performed to evaluate the half-life of mTOR protein. HeLa cells transfected with siIARS2 or siCtrl were treated with CHX (100 µg/mL), and protein samples were collected at 0, 4, 12, 24, and 48 h for western blot analysis (Fig. 3a). Quantitative analysis using ImageJ revealed that mTOR protein degradation occurred more rapidly in the siIARS2 group than in the control group (Fig. 3b). The estimated half-life of mTOR was approximately 10.76 h in siCtrl-treated cells, whereas it was reduced to 5.7 h following IARS2 knockdown. These results demonstrate that depletion of IARS2 promotes mTOR protein degradation.

### The isoleucine-binding domain of IARS2 is required for regulation of mTOR levels and cell proliferation

**Fig. 4:**
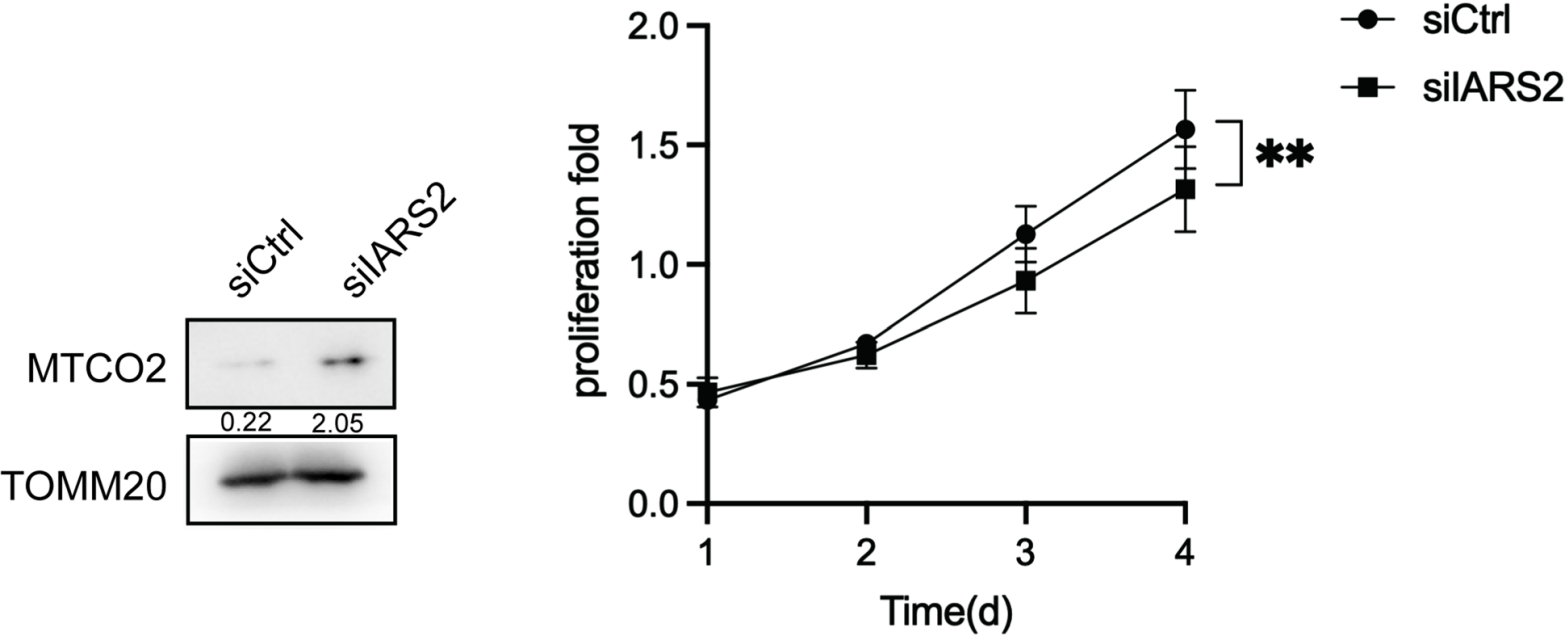
Proliferation inhibition by siIARS2 did not operated through decreasing mitochondrial protein synthesis. The protein expression of MTCO2 in siCtrl group and siIARS2 group i.a. depicted in the left panel, with the corresponding proliferation presented in the right panel.

As IARS2 is a mitochondrial isoleucine-tRNA synthetase^40^, we first investigated whether the inhibitory effects of IARS2 knockdown on cell proliferation were attributable to impaired mitochondrial protein synthesis. The mitochondrial DNA–encoded cytochrome c oxidase subunit II (MTCO2), a representative indicator of mitochondrial protein synthesis^41^, was examined. Western blot analysis showed that MTCO2 protein levels were not reduced in siIARS2-treated cells compared with controls, despite the observed decrease in cell proliferation (Fig. 4). This suggests that suppression of cell proliferation by IARS2 knockdown is independent of mitochondrial protein synthesis.

**Fig. 5:**
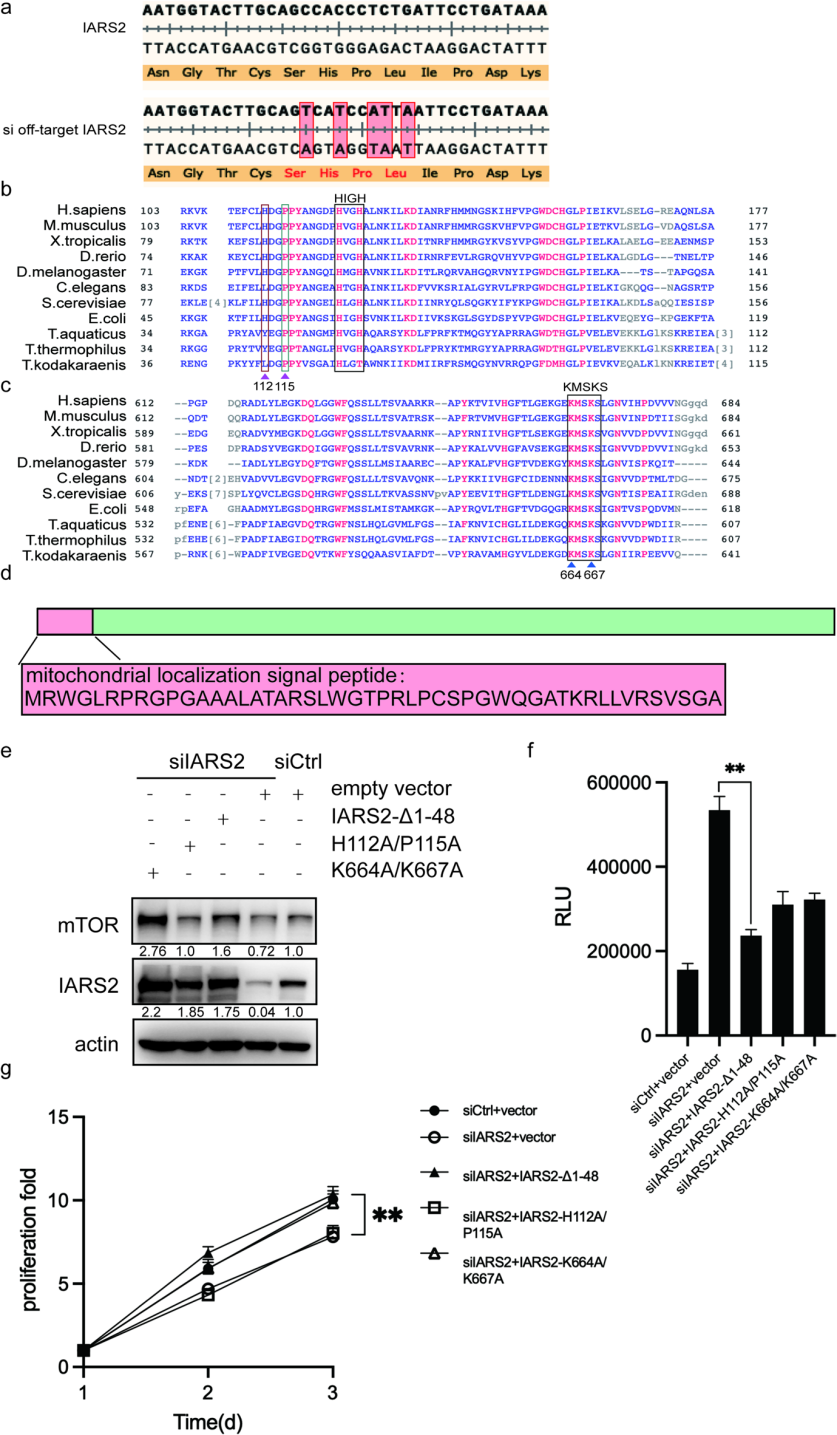
IARS2 function as amnio acids sensors in mTORC1 pathway. (**a**) The sequence alignment of IARS2 and off-target IARS2 plasmid at siIARS2 targeting region. The picture represents the synonymous mutation position and the corresponding amino acids. **(b**-**c)** Primary sequence alignment of IARS2 of various species. The class 1a has conserved HIGH motif important to ATP binding, and KMSKS motif important for tRNA binding. Conserved His and Pro are indicated in red or green frame respectively. Conserved Lys are indicated by blue triangles. **(d)** The sequence of IARS2 mitochondrial localization signal is represented. **(e)** The protein level of mTOR and IARS2 in siCtrl group, siIARS2 group, siIARS2 + H112A/P115A (putative isoleucine binding deficiency variant) or K664A/K667A (putative tRNA binding deficiency variant), IARS2-Δ1-48-flag (mitochondrial localization signal deletion variant) group. **(f)** Caspase3/7 activity result. The Y-axis shows the caspase 3/7 activity, indicated as relative luminescence unit (RLU). **(g)** CCK8 result. The Y-axis represents the absorbance value at 450 nm.

To identify the functional key residue of IARS2 responsible for regulating mTOR and cell viability, a series of IARS2 mutant plasmids resistant to siIARS2-mediated silencing were constructed (Fig. 5a). Sequence alignment across species revealed conserved HIGH (HVGH in IARS2) and KMSKS motifs. Based on previous studies of LARS^10^, a putative isoleucine-binding–deficient mutant was generated by substituting His112 and Pro115 with alanine (H112A/P115A; Fig. 5b). A putative tRNA-binding–deficient mutant (K664A/K667A; Fig. 5c) and a mitochondrial localization signal–deleted mutant (Δ1–48; Fig. 5d) were also constructed.

HeLa cells were co-transfected with siIARS2 and each mutant plasmid. Western blot analysis confirmed successful expression of the mutant IARS2 proteins despite siIARS2 treatment (Fig. 5e). Rescue experiments demonstrated that both the Δ1–48 and K664A/K667A mutants restored mTOR protein levels and cell proliferation while reducing apoptosis (Fig. 5e–g). In contrast, the H112A/P115A mutant suppressed apoptosis but failed to restore mTOR expression or cell proliferation. These results indicate that the putative isoleucine-binding key residues of IARS2 is required for mTORC1-mediated cell proliferation, but is dispensable for apoptosis regulation.

### IARS2 interacts with Rag GTPases

**Fig. 6:**
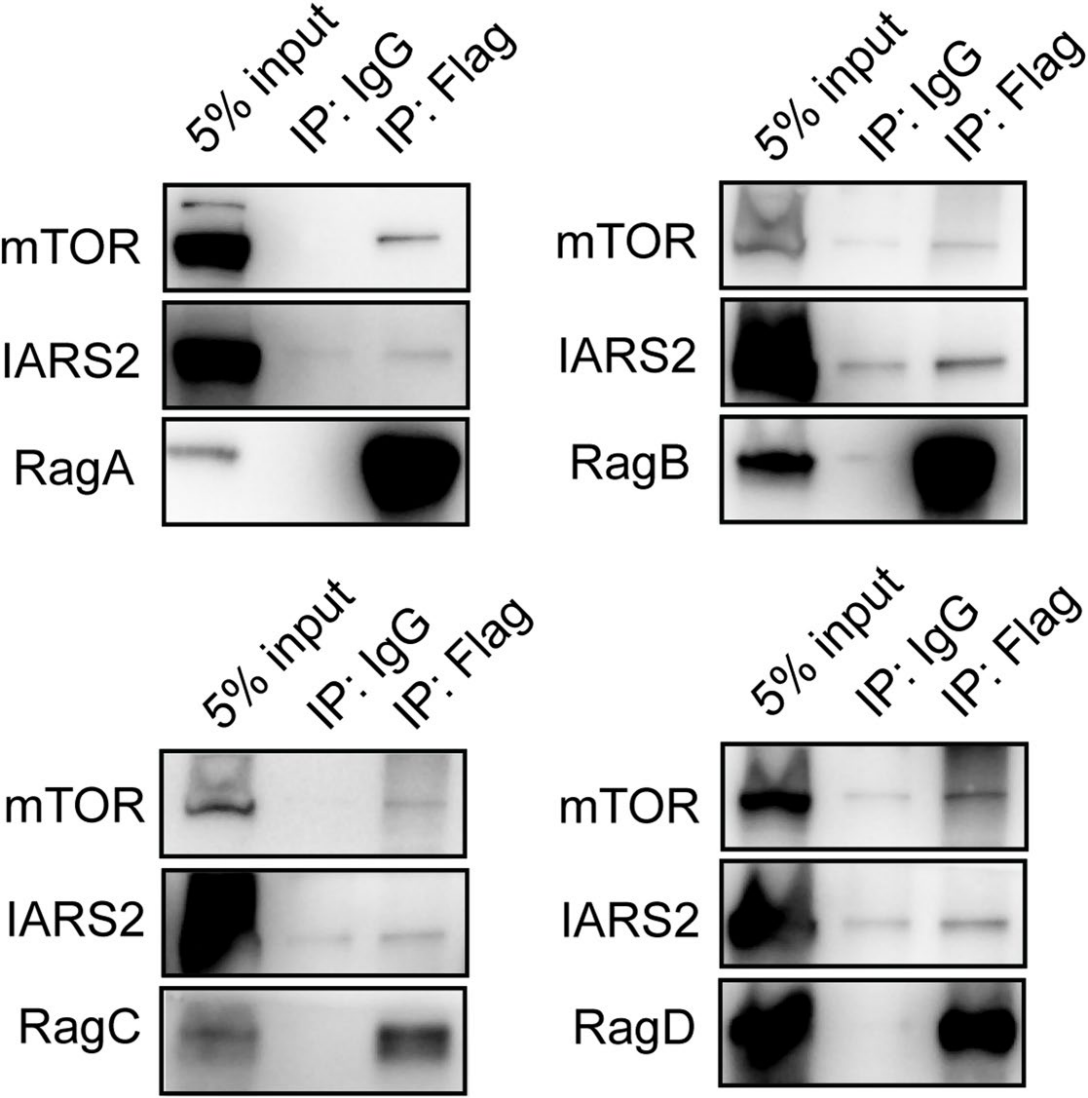
Co-IP of IARS2 with Rags. HeLa cells were transfected with indicated Rag GTPases-flag plasmids and immunoprecipitated with mouse anti-flag primary antibody or normal mouse IgG. Rabbit anti-flag primary antibody and corresponding primary antibody of IARS2 or mTOR are utilized to perform western blot.

Given that IARS2-mediated regulation of mTOR signaling was independent of its tRNA-charging activity, we investigated whether IARS2 interacts with Rag GTPases, which are essential for amino acid–dependent mTORC1 activation. Rag heterodimers recruit mTORC1 to lysosomes in response to amino acid availability, and LARS has been reported to function as a leucine sensor in this process^10,42^. In addition, mitochondrial threonyl-tRNA synthetase (TARS2) has been shown to act as a threonine sensor regulating mTORC1 activation^26^. Co-immunoprecipitation assays revealed that heterodimeric Rag GTPases, particularly RagB and RagD, co-precipitated with endogenous IARS2 and mTOR (Fig. 6). These findings indicate that IARS2 associates with Rag GTPases and may participate in the Rag–mTORC1 complex.

## Discussion

Although IARS2 has been implicated in proliferation across multiple tumor types, the molecular mechanisms underlying its role in tumorigenesis remain poorly understood^12,38^. This study systematically investigated IARS2 function in cervical cancer and uncovered a mechanism distinct from canonical mTOR signaling activation: IARS2 promotes tumor cell proliferation by stabilizing mTOR protein rather than modulating its phosphorylation status, suggesting its role extends beyond the canonical function in mitochondrial translation.

Previous studies have documented IARS2 amplification and overexpression in various cancers, but most remained at the level of phenotypic correlations^43^. Our study integrates clinical data, functional experiments, and molecular mechanisms: high IARS2 expression correlates with poor prognosis in cervical cancer patients (Fig. 1); knockdown experiments confirm its pro-proliferative and anti-apoptotic functions; and importantly, we studied how IARS2 regulates mTOR protein homeostasis.

To preliminarily assess the effect of siIARS2 on apoptosis, flow cytometric analysis was performed using an Annexin V–FITC/PI apoptosis detection kit. The proportion of early apoptotic (Annexin V–positive) cells increased from 4.82 ± 0.20% in the siCtrl group to 6.12 ± 0.28% in the siIARS2 group (*p* < 0.05; Fig. S1). Although the absolute increase was modest, this result indicates a statistically significant shift toward apoptotic initiation following IARS2 silencing. It should be noted that Annexin V–based assays rely on phosphatidylserine (PS) externalization, a transient early apoptotic event, and may therefore underestimate apoptosis when genetic perturbations primarily affect upstream signaling rather than extensive membrane disruption. Accordingly, to achieve a more sensitive and mechanism-oriented quantification of apoptotic activation, we subsequently employed a caspase-3/7 activity–based assay.

A key finding is the mode by which IARS2 regulates mTOR. IARS2 knockdown substantially reduced total mTOR protein levels without proportionally decreasing Ser2448 phosphorylation, indicating that IARS2 primarily regulates mTOR abundance rather than kinase activity. Cycloheximide chase experiments showed that IARS2 depletion shortened mTOR half-life, demonstrating that IARS2 maintains mTOR stability by limiting degradation (Fig. 2a). This “protein homeostasis-based regulation” is distinct from extensively studied “signaling activation-based regulation.” However, the precise mechanism by which IARS2 regulates mTOR protein degradation was not investigated in the present study. The protein degradation pathways within eukaryotic cells include the proteasome and lysosome^44,45^. Previously, Sung-Hoon Kim found that IARS2 could interact with LAMTOR1 on the lysosome surface^26^. LAMTOR1 is a key component of the Ragulator complex^46^, that primarily recruits the mTORC1 complex to the lysosome surface and enables mTORC1 complex to exert regulatory functions^24^. Therefore, we speculated that IARS2 may affect mTOR protein degradation through lysosomal pathway, and further experiments were needed to verify this hypothesis in the future.

Downstream of mTORC1, we identified eIF4E as a key mediator of IARS2’s biological effects. Studies have found that overexpression of eIF4E is closely associated with the persistent presence of high-risk HPV^47^, that emphasized the importance of eIF4E in HPV-related cervical cancer. We found that IARS2 knockdown reduced mTOR protein levels, leading to decreased eIF4E expression and ultimately resulting in impaired proliferation and enhanced apoptosis. eIF4E rescue experiments partially reversed these phenotypes, supporting its functional role in the IARS2-mTOR axis (Fig. 2e, Fig. S2**e**). In the siIARS2 + eIF4E rescue group, eIF4E protein levels were significantly elevated compared with the siCtrl group, confirming successful restoration. Consistently, the CCK-8 and Caspase-3/7 Glo assay results shown in Fig. 2e indicate that eIF4E re-expression partially counteracted the inhibitory effects of IARS2 silencing, further supporting the conclusion that eIF4E is an important downstream effector of IARS2. It should be noted, however, that eIF4E possesses intrinsic pro-proliferative and anti-apoptotic activities. Therefore, we cannot exclude the possibility that eIF4E overexpression exerts effects that are at least partially independent of IARS2. This limitation could be addressed in future studies by including eIF4E-alone overexpression controls.

To elucidate the relation of IARS2 and mTORC1 in cervical cancer, we studied the functional key residues of IARS2 in regulating cervical cancer. IARS2 mainly contain mitochondrial localization signal, amino acids-binding domain, and tRNA-binding domain. Our results showed the key residues for amino acid-binding of IARS2 was required for regulating mTOR. As we all know, mTORC1 can regulate cell proliferation and metabolism by sensing intracellular nutrients, such as amino acids^48^. Previous studies proposed that LARS and TARS2 could serve as amino acids sensors involving in the activation of mTORC1, providing novel roles for aminoacyl-tRNA synthetases beyond their classical functions. But this study indirectly suggested the same function of IARS2, in the future isoleucine starvation experiments will be needed for demonstrating the necessary of IARS2 and Ile in the mTORC1. Meanwhile we found the silencing of IARS2 put few impacts on mitochondrial protein and the reason was unclear (Fig. 4). This suggests IARS2’s pro-tumorigenic function may be largely independent of its mitochondrial translation role. Possible explanations include residual protein sufficiency for basal translation, functional redundancy in the system, or the existence of a non-mitochondrial IARS2 pool participating in signaling.

In previous studies on the amino acid-dependent mTORC1 pathway, Ras-related GTP-binding proteins (Rags) have played a crucial role in the activation of mTORC1^24^. Sung-Hoon kim et al. proposed that the pathway Thr/TARS2/RagA-C/mTORC1, among TARS2 and RagA-C as bridges to regulating mTORC1^26^. TARS2 facilitated GTP hydrolysis by RagC and GTP loading by RagA, converting the silent RagA^GDP^-RagC^GTP^ dimer to an active state. Subsequently, mTORC1 was recruited to the lysosomal surface by the RagA^GTP^-RagC^GDP^ and activated^26^. Inspired by this, we initially demonstrated through co-immunoprecipitation experiments that all four isoforms of Rag GTPase in cervical cancer cells can interact with IARS2. However, it should be noted that the co-immunoprecipitation assay cannot determine whether the interaction is direct or indirect, nor can it quantify the strength of the interaction. The specific isoforms that serve as the bridging factors between IARS2 and mTORC1 remain unclear. However, this also indirectly validates the potential role of IARS2 as an amino acid sensor in the amino acid/mTORC1 pathway. In the future, we will further investigate the relationship between GTP/GDP and Rags-IARS2.

In conclusion, we investigated the role of IARS2 in regulating proliferation and apoptosis of HeLa cells and proposed that IARS2 served as Ile sensor in Rags-mTORC1 complex. This revealed the specific mechanism of oncogene IARS2 in regulating the proliferation and apoptosis of cervical cancer cells, providing a novel potential therapeutic target and theoretical basis for the treatment of cervical cancer. Cancers typically have higher requirements for essential amino acids^49^, limiting proteins or amino acids intaking and starving cancers therapy is one hotspot of cancer treatment^50^. The essential amino acid isoleucine plays a certain role in the activation of mTORC1 in cervical cancer cells. Therefore, isoleucine, as an initial stimulatory factor, could potentially be used in restricted isoleucine diets as a novel dietary therapy to aid in the treatment of cervical cancer in the future. Additionally, previous studies have shown that amino acid starvation therapy can enhance the effectiveness of conventional chemotherapy^51^, providing a new avenue for combined therapy in the treatment of cervical cancer.

## Supplementary Information

Below is the link to the electronic supplementary material.

Supplementary Material 1

Supplementary Material 2

Supplementary Material 3

## Data Availability

Public data were obtained from the following websites: GEPIA (https://GEPIA.cancer-PKU.cn/). Most of the results of the current study appear in the article or as supplementary materials. The sequence datasets of plasmids generated during the current study are available in https://www.addgene.org/browse/article/28252609/ . In case of reasonable request, the corresponding author can provide further details regarding the data. Generated Statement: The raw data supporting the conclusions of this article will be made available by the authors, without undue reservation.
