# Supplementary material for: Mechanism of isoleucyl-tRNA synthetase 2 regulating proliferation and apoptosis of cervical cancer cells
Source: Sci Rep. 2026 Mar 2;16:11578. doi: 10.1038/s41598-026-41218-7 (PMC13056935; doi:10.1038/s41598-026-41218-7)
Supplement: Supplementary file 2 — Supplementary Material 2 [file 41598_2026_41218_MOESM2_ESM.docx]

Supplementary Fig. S2a

p-mTOR Ser2448 (regular ECL)


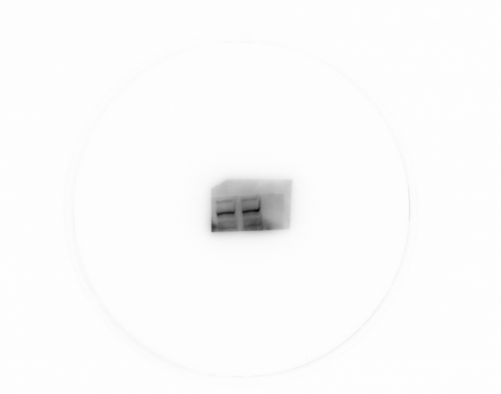


mTOR（14s/10s）(regular ECL)


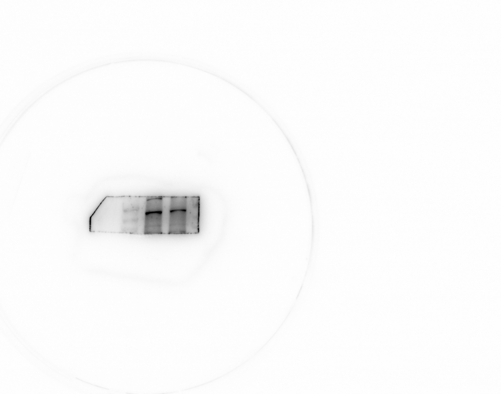

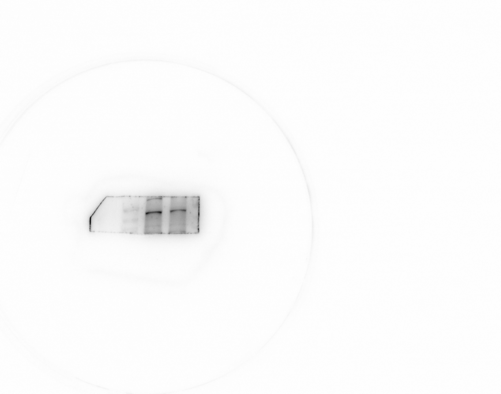


IARS2 (regular ECL)


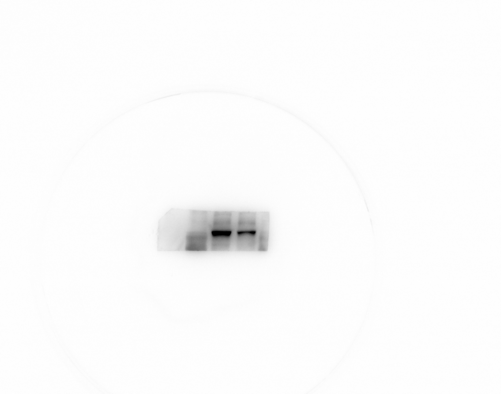


Actin (regular ECL)


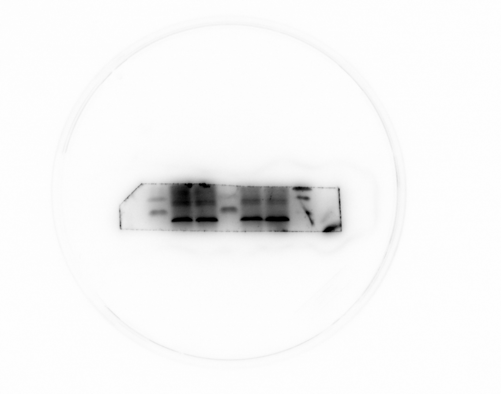


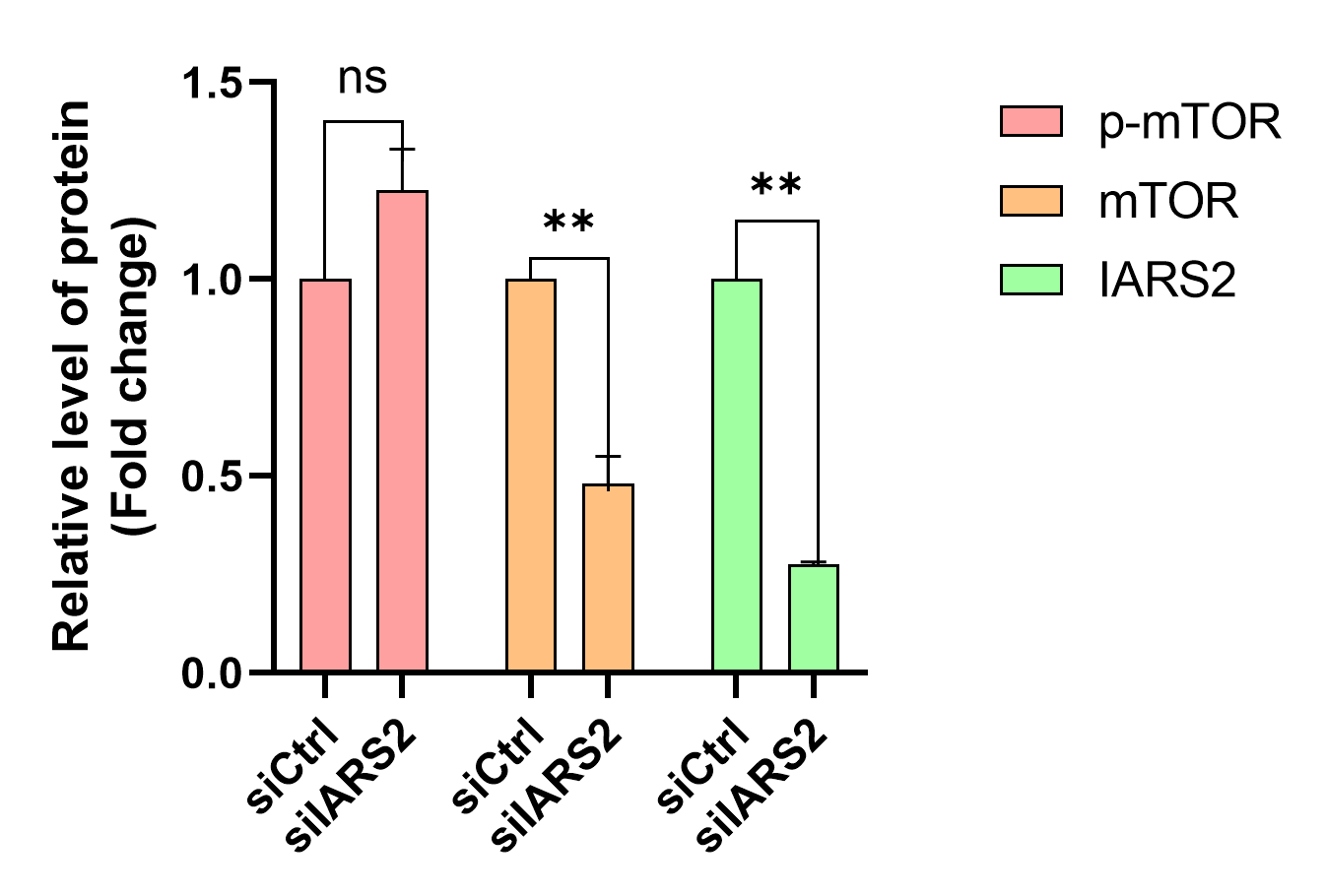


Supplementary Fig. S2a. All images on the left are the original images used in the article, while the images on the right represent the same images with different exposure levels. The bar graph shows the relative expression levels of proteins. All values were normalized to the control group. Data are from two independent biological replicates. p < 0.01 denotes **.

Supplementary Fig. S2b

mTOR (ultra-sensitive ECL)


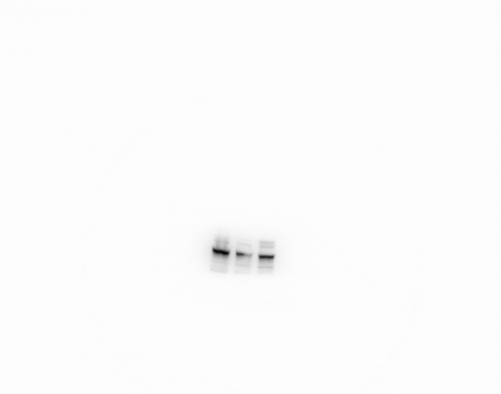


IARS2 (ultra-sensitive ECL)


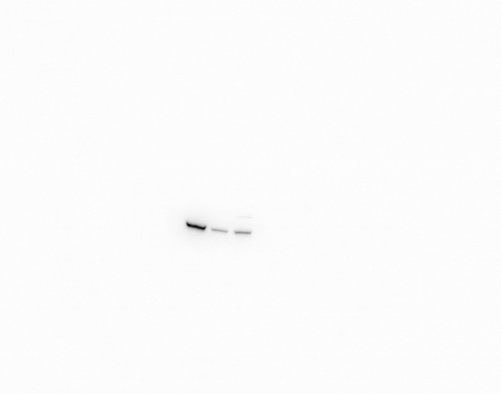


eIF4E (ultra-sensitive ECL)


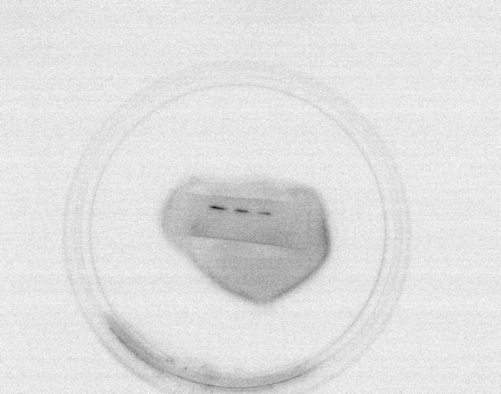


Actin (ultra-sensitive ECL)


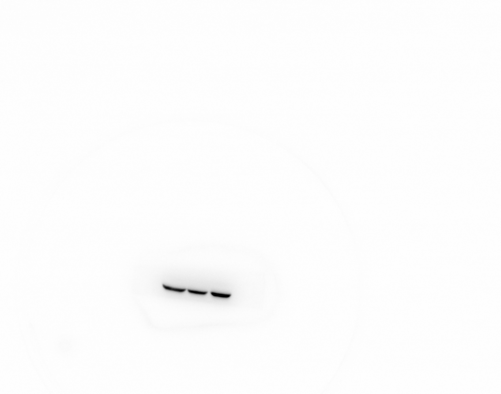


Supplementary Fig. S2e

IARS2 (ultra-sensitive ECL)


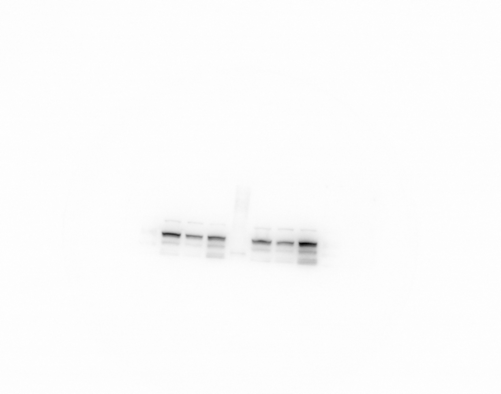


eIF4E (ultra-sensitive ECL)


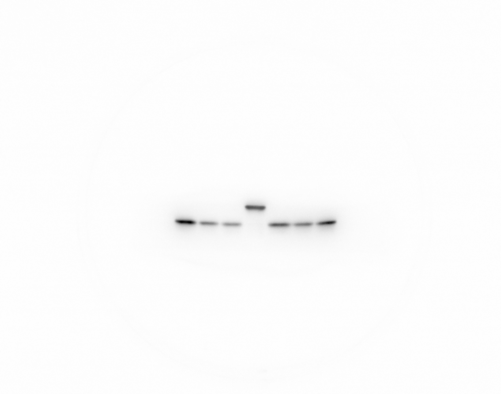


Actin (ultra-sensitive ECL)


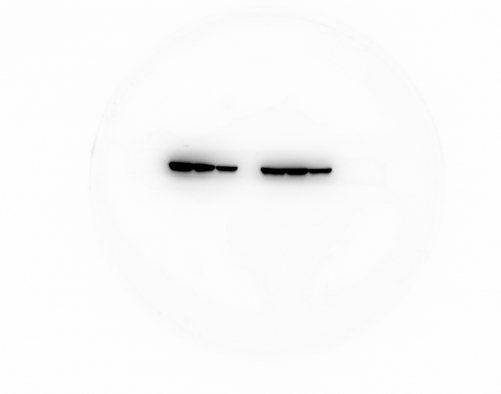


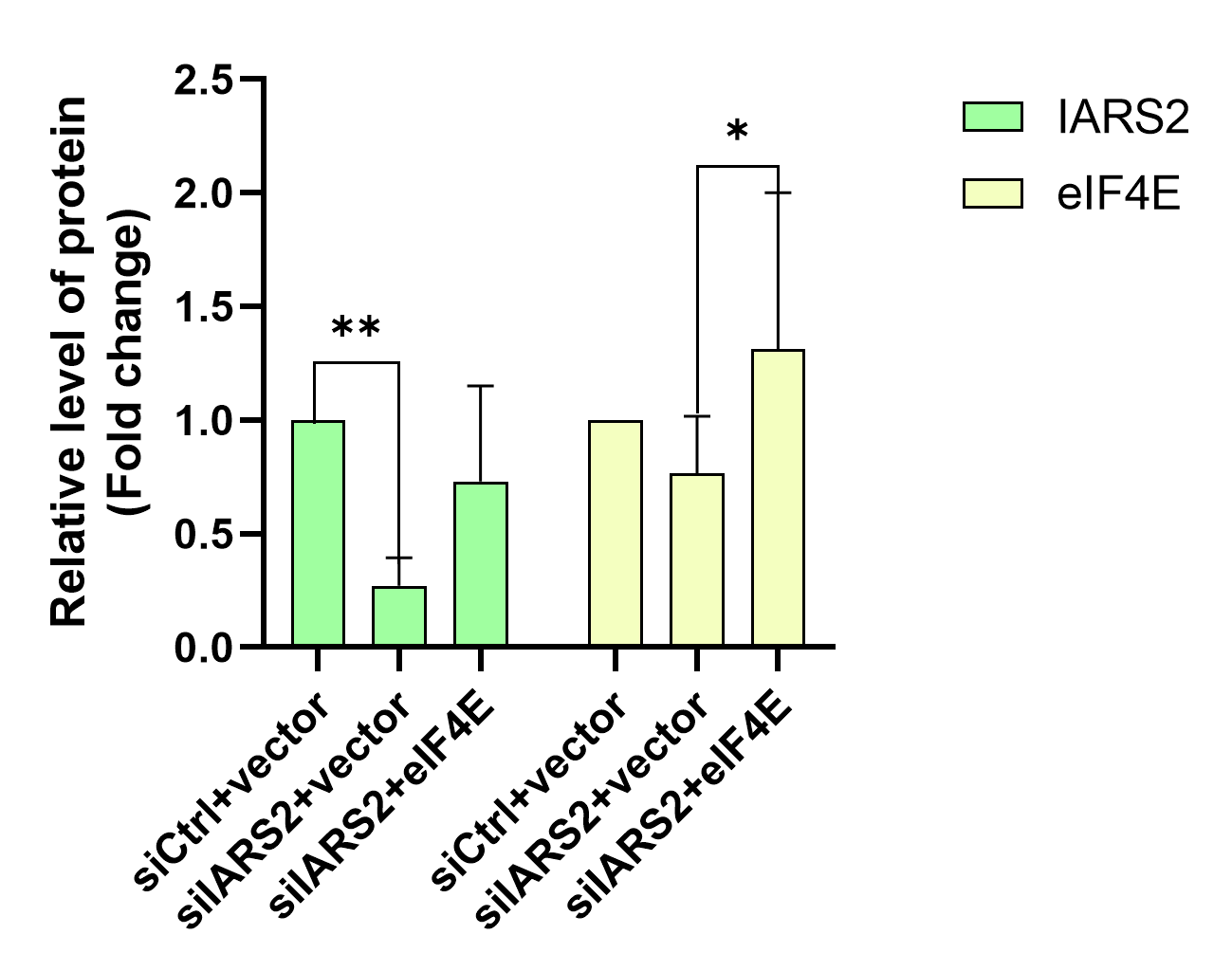


Supplementary Fig. S2e. The bar graph shows the relative expression levels of proteins. All values were normalized to the control group. Data are from three independent biological replicates. p < 0.05 denotes *.

Supplementary Fig. S3a

mTOR (ultra-sensitive ECL)


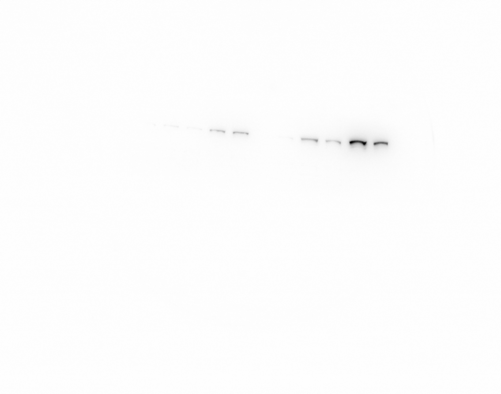


IARS2 (ultra-sensitive ECL)


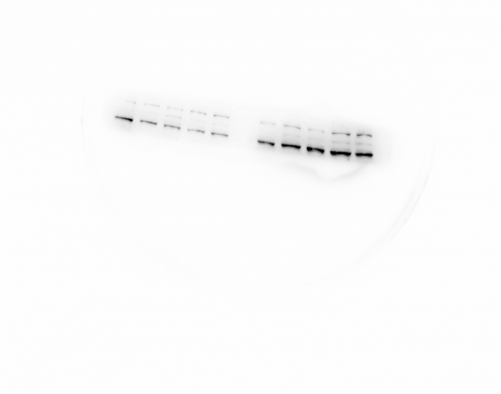


Actin (ultra-sensitive ECL)


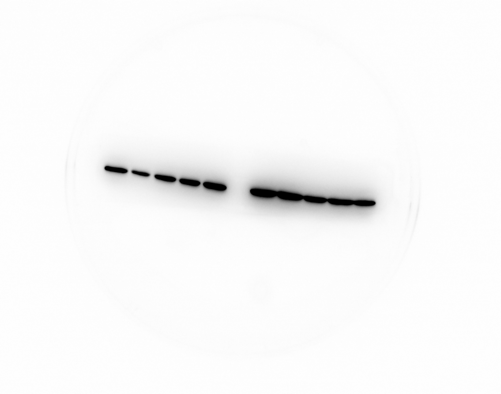


Supplementary Fig. S4

Mtco2 (ultra-sensitive ECL)


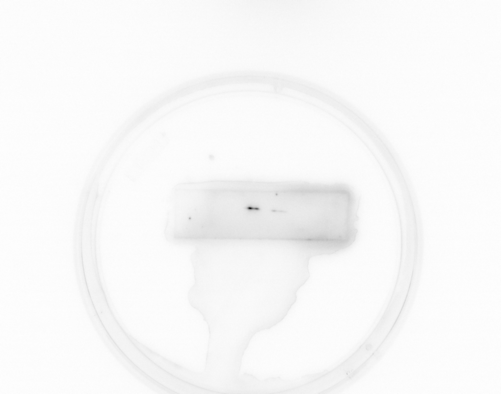


Tomm20 (ultra-sensitive ECL)


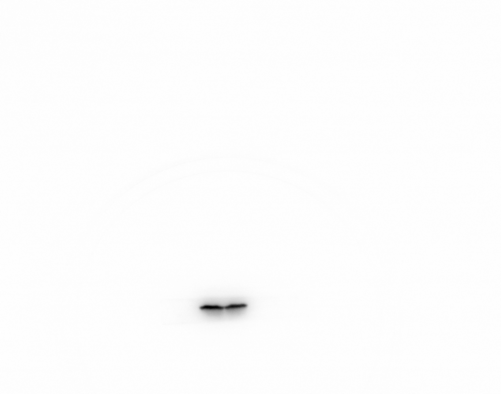


Supplementary Fig. S5e

mTOR (45s/1min) (ultra-sensitive ECL)


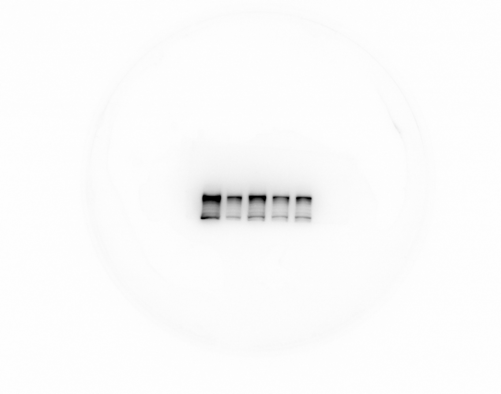

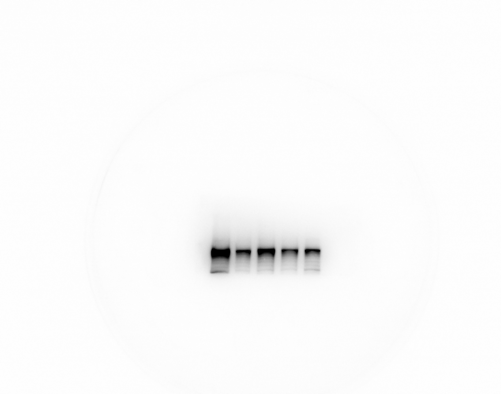


IARS2 (ultra-sensitive ECL)


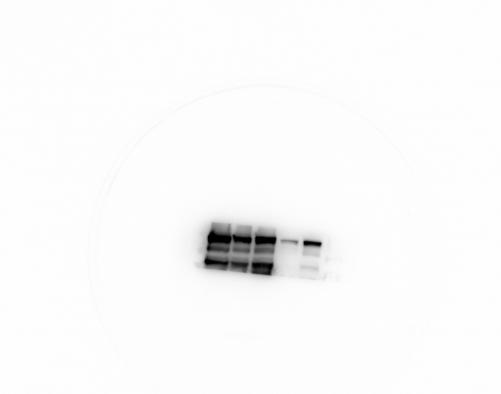


Actin (40s/1min10s) (ultra-sensitive ECL)


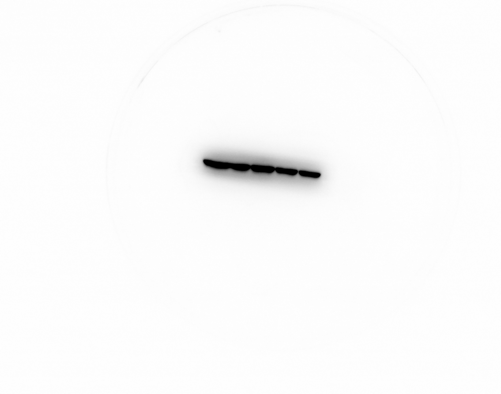

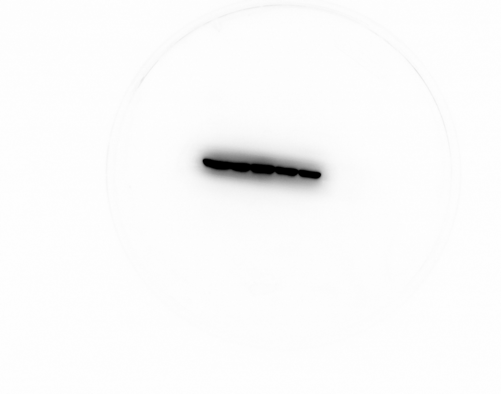


Supplementary Fig. S5. All images on the left are the original images used in the article, while the images on the right represent the same images with different exposure levels.

Supplementary Fig. S6

mTOR (ultra-sensitive ECL)


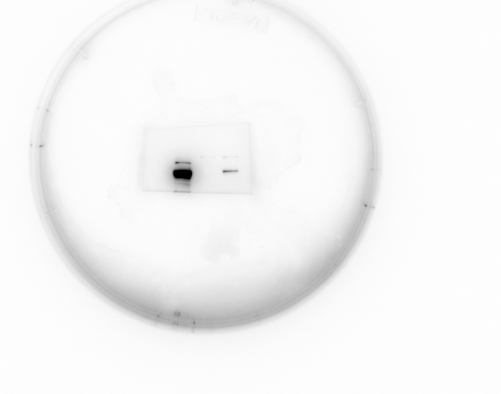


IARS2 (ultra-sensitive ECL 15s/regular ECL 30s)


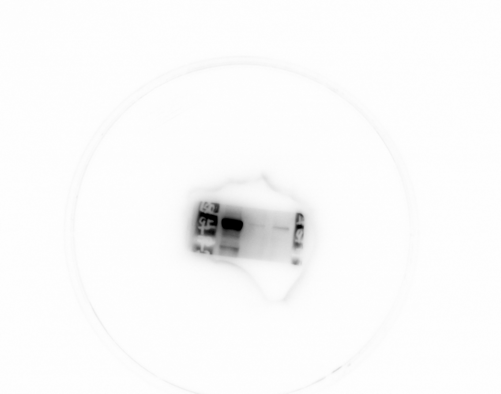

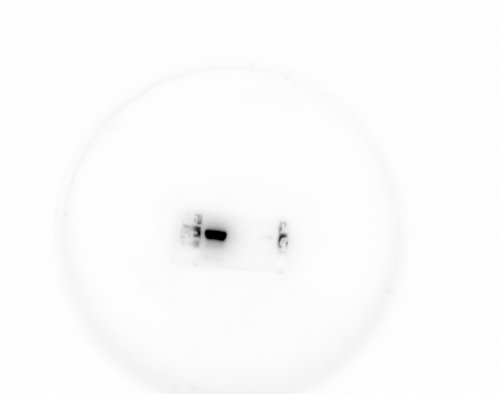


RagA (ultra-sensitive ECL)


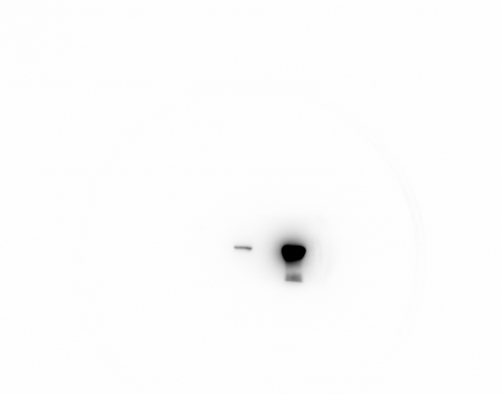


Supplementary Fig. S6

mTOR (10s/20s) (regular ECL)


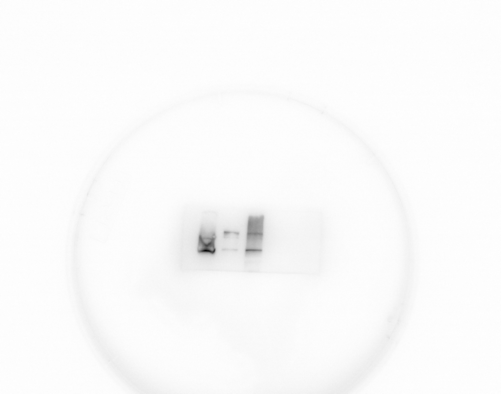

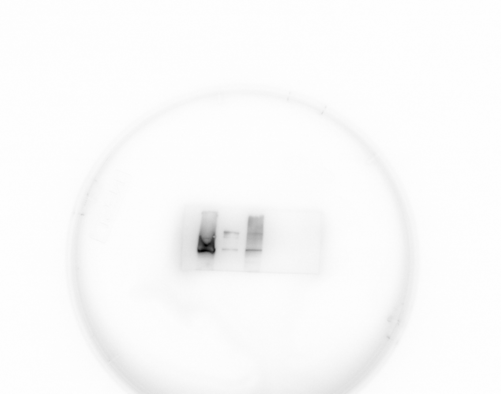


IARS2 (10s/15s) (regular ECL)


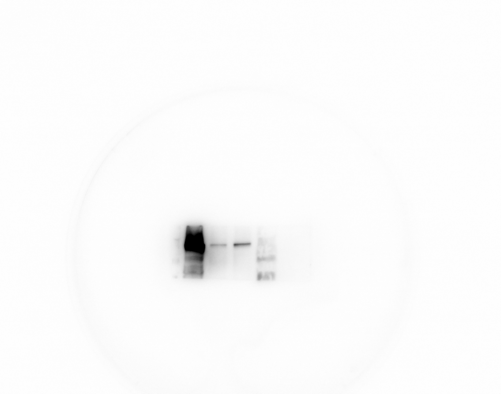

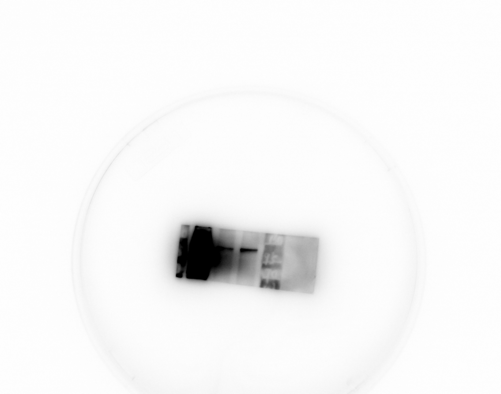


RagB (regular ECL)


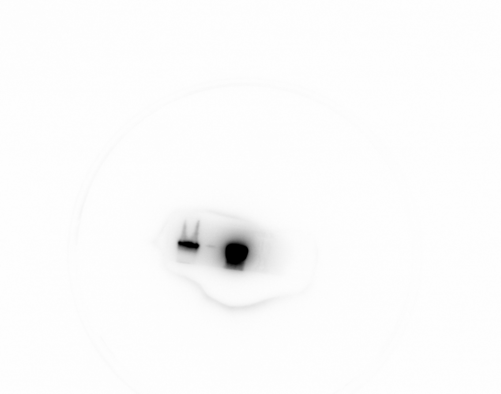


Supplementary Fig. S6

mTOR (regular ECL)


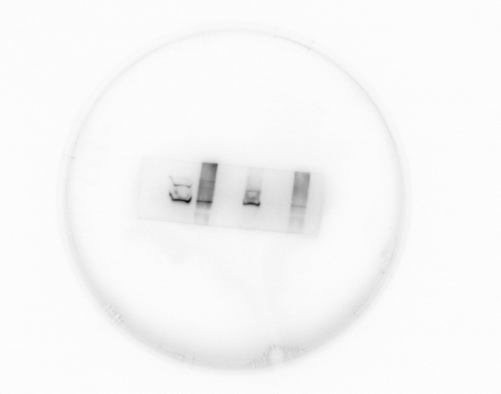


IARS2 (18s/10s) (regular ECL)


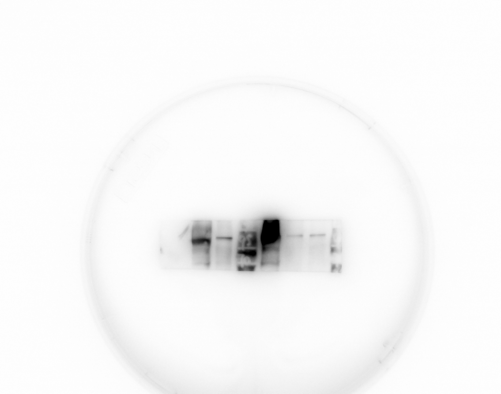

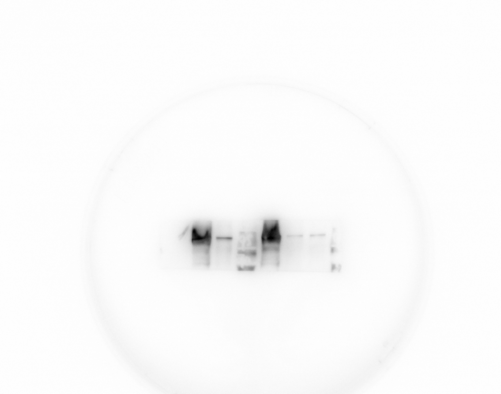


RagC (1min/30s) (regular ECL)


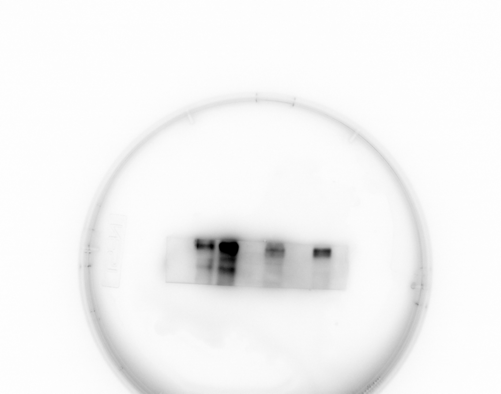

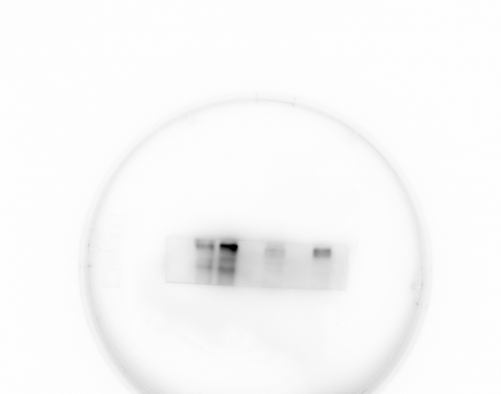


Supplementary Fig. S6

mTOR (ultra-sensitive ECL 10s/regular ECL 20s)


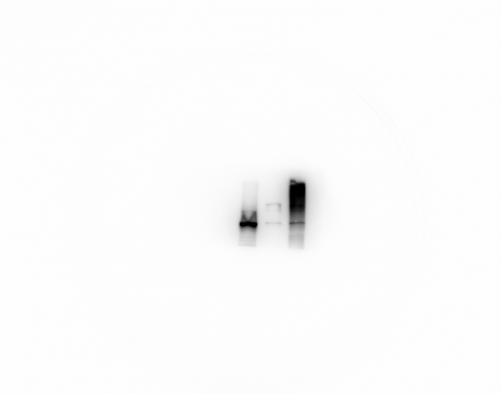

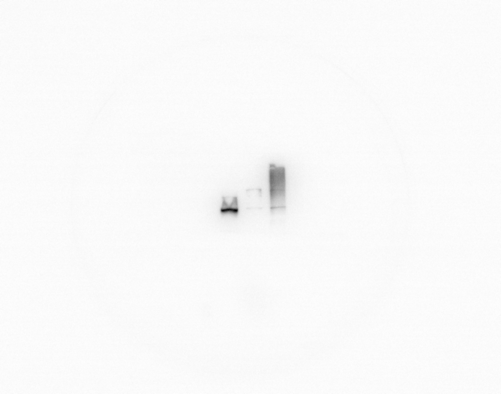


IARS2 (ultra-sensitive ECL 30s/regular ECL 30s)


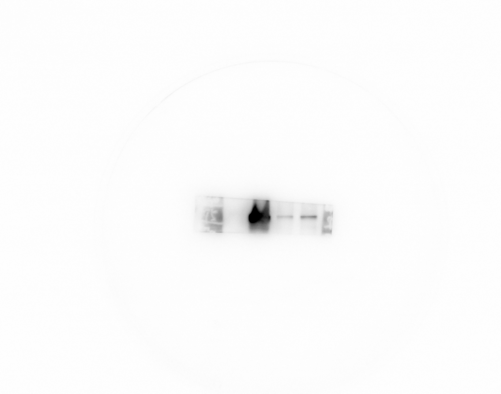

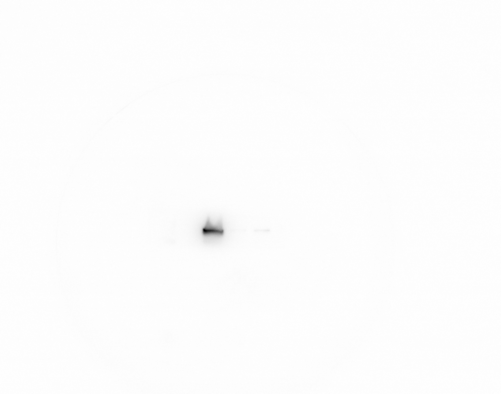


RagD (1min30s/1min) (ultra-sensitive ECL)


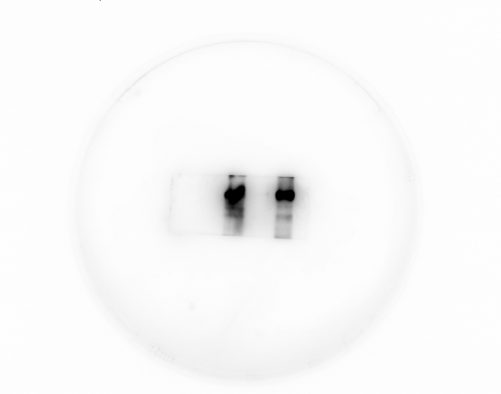

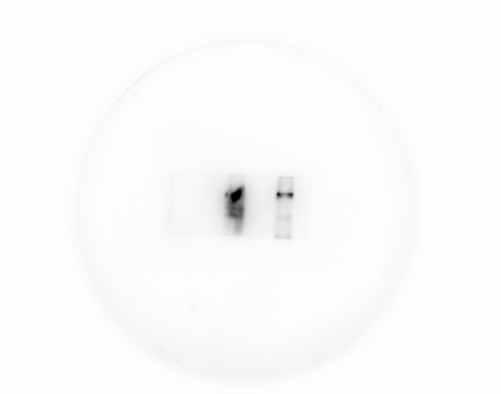


Supplementary Fig. S6. All images on the left are the original images used in the article, while the images on the right represent the same images with different exposure levels.
