# Supplementary material for: Mechanism of isoleucyl-tRNA synthetase 2 regulating proliferation and apoptosis of cervical cancer cells
Source: Sci Rep. 2026 Mar 2;16:11578. doi: 10.1038/s41598-026-41218-7 (PMC13056935; doi:10.1038/s41598-026-41218-7)
Supplement: Supplementary file 3 — Supplementary Material 3 [file 41598_2026_41218_MOESM3_ESM.docx]

**Supplementary table S1. The sequence of primers for constructing plasmids in this study.**

| **Plasmid** | **Primer** | **Sequence** |
| --- | --- | --- |
| pcDNA3.1-IARS2 | IARS2-SalkzATG-F | TAGAGTCGACCGGACCATGCGTTGGGGGCTG |
|  | IARS2-EcoRITAG-R | TGTCGAATTCCTATTTTCCACTGACAACTTCTGCACATC |
| pcDNA3.1-eIF4E | eIF4E-Xho-F | ATCTCTCGAGATGGCGACTGTCGAACCGGAAACC |
|  | eIF4E-HindTAA-R | TGTCAAGCTTAAACAACAAACCTATTTTTAGTGGTGGAG |
| pcDNA3.1-IARS2-tb | IARS2-tb-F | TGGTACTTGCAGCCACCCTCTGATTCCTGATAAA |
|  | IARS2-tb-R | TTTATCAGGAATCAGAGGGTGGCTGCAAGTACCA |
|  | pcDNA3.1Pme-F | GGAGACCCAAGCTGGCTAGCGTTT |
|  | pcDNA3.1Pme-R | ACAGTCGAGGCTGATCAGCGGTTT |
| pcDNA3.1-IARS2-H112A/P115A | IARS2-tb-H112/P115A-F | CAGAATTTTGCCTTGCTGATGGAGCTCCTTATGCAA |
|  | IARS2-tb-H112/P115A-R | TTGCATAAGGAGCTCCATCAGCAAGGCAAAATTCTG |
| pcDNA3.1-IARS2-K664A/K667A | IARS2-tb-K664/K667A-F | GAGAAAAGGGAGAAGCcATGTCCGCCTCTCTTGGGAATGT |
|  | IARS2-tb-K664/K667A-R | CAATTCCCAAGAGAGGCGGACATGGCTTCTCCCTTTTCTC |
| pcDNA3.1-flag-IARS2-Δ1-48 | IARS2-SalkzATG-Δ1-48-F | TAGAGTCGACCGGACCATGAGTAACCACCAGCCGAACTCGA |
|  | IARS2-EcoRITAG-R | TGTCGAATTCCTATTTTCCACTGACAACTTCTGCACATC |

**Supplementary table S2. Addgene ID of plasmids constructed in this work.**

| **Plasmid** | **Addgen ID** |  |
| --- | --- | --- |
| pcDNA3.1-IARS2 | 232337 | https://www.addgene.org/232337/ |
| pcDNA3.1-eIF4E | 232336 | https://www.addgene.org/232336/ |
| pcDNA3.1-IARS2-tb | 232340 | https://www.addgene.org/232340/ |
| pcDNA3.1-IARS2-H112A/P115A | 232338 | https://www.addgene.org/232338/ |
| pcDNA3.1-IARS2-K664A/K667A | 232339 | https://www.addgene.org/232339/ |
| pcDNA3.1-flag-IARS2-Δ1-48 | 232341 | https://www.addgene.org/232341/ |

| **Gene** | **Primer** | **Sequence** |
| --- | --- | --- |
| IARS2 | IARS2-q-F | TGGACCTCCTTATGCAAACGG |
|  | IARS2-q-R | GGCAACCCATGACAATCCCA |
| eIF4E | eIF4E-SybrG-F | CGGAATCTAATCAGGAGGTTGC |
|  | eIF4E-SybrG-R | GATCAGCCGCAGGTTTGC |
| GAPDH | GAPDH-SybrG2-F | CCCACTCCTCCACCTTTGACG |
|  | GAPDH-SybrG2-R | CACCACCCTGTTGCTGTAGCCA |

**Supplementary table S3. The sequence of primers for qRT-PCR in this study.**

**Supplementary Fig. S1**

**b**

**a**


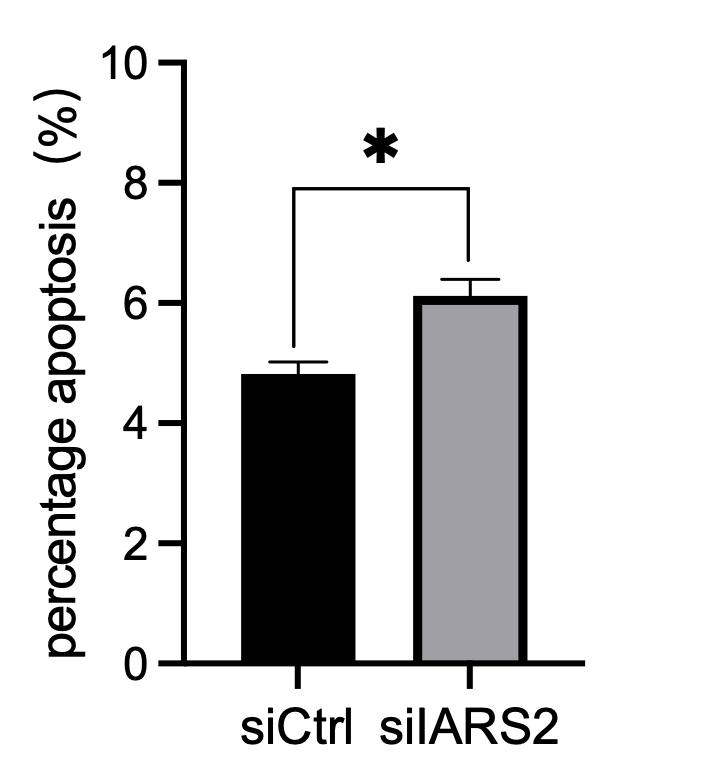


Supplementary Fig. S1. (a) The pictures on above show the results of flow cytometry. After cells stained with Annexin V-APC, flow cytometry is used to analyze the fluorescence of cells. Q3 represents the cells in the early stage of apoptosis.

(b) Histograms represent the quantification of apoptotic cells counts in different groups.
